# Clinical features and prognostic factors of anti‐melanoma differentiation‐associated gene 5 antibody‐positive dermatomyositis with rapidly progressive interstitial lung disease in Chinese patients

**DOI:** 10.1002/iid3.882

**Published:** 2023-06-14

**Authors:** Li Lian, Jing‐jing Tong, Sheng‐qian Xu

**Affiliations:** ^1^ Department of Rheumatology and Immunology The First Affiliated Hospital of Anhui Medical University Hefei China

**Keywords:** anti‐MDA5 antibody, dermatomyositis, rapidly progressive interstitial lung disease

## Abstract

**Objective:**

The objective of this study is to investigate clinical features and prognostic factors of antimelanoma differentiation‐associated gene 5 (anti‐MDA5)‐positive dermatomyositis with rapidly progressive interstitial lung disease (RP‐ILD) in Chinese patients.

**Methods:**

Clinical features and prognostic factors of patients with newly diagnosed or recurrent dermatomyositis patients were retrospectively analyzed. All patients were divided into the anti‐MDA5‐positive or negative dermatomyositis, and with or without RP‐ILD groups. Clinical features and prognostic factors were statistically compared among different groups.

**Results:**

The serum ferritin (SF) levels (1500.0 [658.80, 1844.0]) and γ‐glutamyl transpeptidase (γ‐GT) (125.5 [61.0, 232.0] vs. 28 [16.0, 41.0], *Z* = 5.528; *p* < .001) were markedly higher, and phosphocreatine myoenzyme (CK) (73.0 [42.0, 201.0] vs. 1333.0 [79.0, 8000.0], *Z* = −2.739, *p* = .006), serum albumin level (32.51 ± 5.23 vs. 35.81 ± 5.88, *t* = −2.542, *p* = .013), and lymphocyte count (0.80 ± 0.36 vs. 1.45 ± 0.77, *t* = −4.717, *p* < .001) were lower than those in anti‐MDA5‐negative counterparts. Among patients with anti‐MDA5 antibody (Ab) with RP‐ILD, the SF level (1531.0 [1163.8, 2016.5] vs. 584.9 [564.8, 1042.5], *Z* = 2.664, *p* = .008), γ‐GT (134.0 [81.0, 204.5] vs. 123.0 [76.0, 189.0], *Z* = 3.136, *p* = .002) and positive rate of anti‐RO‐52 Ab (90.9% vs. 50.0%, *χ* ^2^ = 7.222, *p* = .013) were higher and lymphocyte count (0.79 ± 0.38 vs. 1.32 ± 0.74, *t* = −3.025, *p* = .029) was lower than those in their counterparts without RP‐ILD. The SF level of anti‐MDA5 nonsurvivors (1544 [1447.32, 2089.0] vs. 584.9 [515.7, 1500.0], *Z* = 2.096, *p* = .030), anti‐RO‐52 Ab‐positive rate ([16/18, 88.9%] vs. [9/16, 56.2%], *χ* ^2^ = 4.636, *p* = .031) were higher than those in survivors. Lymphocytopenia was a risk factor for RP‐ILD and death of patients with anti‐MDA5‐positive dermatomyositis. The area under receiver operating characteristic curve was 0.888 (95% confidence interval: 0.756, 1.000; *p* < .001), the sensitivity was 85.7%, the specificity was 93.8%, and Youden's index was 0.795.

**Conclusions:**

Anti‐MDA5‐positive dermatomyositis patients are prone to developing RP‐ILD. Declined lymphocyte count is a critical risk factor for RP‐ILD, probably acting as a simple and effective predictor for Chinese patients with anti‐MDA5‐positive dermatomyositis.

## Graphical abstract

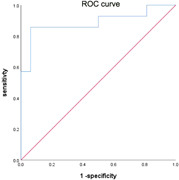

## 1 INTRODUCTION

Dermatomyositis is an autoimmune, idiopathic inflammatory disease affects muscles, skin, lungs, and other organs. Clinical features are diverse and heterogeneous. The antimelanoma differentiation‐associated gene 5 (anti‐MDA5) antibody (Ab) was first discovered in a Japanese patient cohort with clinically amyopathic dermatomyositis (CADM) in 2005.^1^ Follow‐up studies have demonstrated that dermatomyositis patients with positive Abs are more likely to show CADM and prone to rapidly progressive interstitial lung disease (RP‐ILD).^2^, ^3^, ^4^ Furthermore, these patients are resistant to various traditional treatments with a high mortality rate within 3–6 months.^2^, ^5^ Although multiple prognostic factors have been reported in different cohorts, including serum ferritin (SF),^3^, ^4^ Krebs von den Lungen‐6 (KL‐6),^6^ γ‐glutamyl transpeptidase (γ‐GT),^7^ and lactate dehydrogenase,^8^ and so on. In 2022, Coutant et al.^9^ analyzed the serum samples of 29 anti‐MDA5 dermatomyositis (DM) patients by indirect immunofluorescence on Hep‐2 cells, to identify patterns associated with poor outcome. They found that specific MDA5 pattern was associated with a higher risk to develop interstitial lung disease.^9^ Besides, Ishikawa et al.^10^ have demonstrated a positive correlation between serum interferon (IFN)‐γ levels and the computed tomography ground glass opacity scores, indicating that IFN‐γ may play an important role in the lung pathophysiology of DM patients positive for MDA5.

However, some of the predictors are not universally accessible or widely carried out. Thus, simple, feasible, and robust prognostic biomarkers are still required for anti‐MDA5 Ab‐positive dermatomyositis patients. In this study, we retrospectively analyzed the prognostic biomarkers of anti‐MDA5 Ab‐positive dermatomyositis patients admitted to Department of Rheumatology and Immunology, and potential prognostic factors of RP‐ILD and mortality were identified, aiming to explore convenient and feasible predictive indicators, and improve the survival and prognosis of anti‐MDA5 Ab‐positive dermatomyositis patients.

## 2 MATERIALS AND METHODS

### 2.1 Baseline data

From January 2018 and August 2022, 78 patients diagnosed with dermatomyositis according to Bohan and Peter^11^ criteria, and 2018 DM classification criteria of ENMC (European Neuro Muscular Centre International Workshop)^12^ were enrolled in this retrospective study. The exclusion criteria were patients with infections, cancers, and/or other connective tissue diseases and/or pre‐existing heart and lung conditions. RP‐ILD was defined as displaying two or more of the following within 3 months: (1) dyspnea exacerbation; (2) an increase in parenchymal abnormality on high‐resolution computed tomography (HRCT) scan; and (3) one of the following physiological changes: >10% decrease in vital capacity or >1.33 kPa decrease in arterial oxygen tension (PaO_2_).^13^ The study procedures were approved by the ethnics committee of the First Affiliated Hospital of Anhui Medical University (PJ‐20230338).

### 2.2 Data collection

Clinical data, including patient demographics,******** clinical manifestations, laboratory data, auto‐Abs, treatments, and outcomes were collected for all patients by reviewing clinical records. All data were recorded based on the first outpatient in our cohort. ILD was diagnosed by imaging specialists and rheumatologists based on clinical symptoms, physical examination findings, and HRCT imaging findings, and RP‐ILD was diagnosed according to the criteria as previously described.^13^ Anti‐MDA5 Ab and KL‐6, surfactant protein‐D (SP‐D) were measured using an enzyme immunoassay (Medical & Biological Lab‐oratories).

### 2.3 Statistical analysis

Statistical analysis was performed with SPSS version 22.0 (SPSS). The Mann–Whitney *U* test or paired‐sample *t* test was used to analyze continuous data and *χ* ^2^ test for the categorical data. Multivariate logistic regression analysis was adopted to determine the predictors of death. The prediction was quantified by the odds ratio with 95% confidence interval (95% CI) and *p* < .05 was considered statistically significant. Receiver operating characteristic (ROC) analysis was performed to compare the predictive performance of lymphocyte. Alternative cutoff point was calculated by the Youden's index.

## 3 RESULTS

### 3.1 Characteristics of study participants at diagnosis

A total of 78 dermatomyositis patients were enrolled in this study, 40 male and 38 female, aged 53.59 ± 13.10 years on average, with a mean disease duration of 4.39 ± 5.88 months. Of the 78 patients, 34 (43.6%) were positive for anti‐MDA5 Ab and 44 cases were negative for anti‐MDA5 Ab, including 25 cases of antisynthetase Ab, 7 cases of anti‐Mi‐2 Ab, 3 cases of anti‐SRP Ab, 6 cases of TIF1‐g Ab, and 3 cases of anti‐SAE Ab, respectively. Among 78 patients, 62 (62/78,79.45%) were complicated with interstitial pneumonia and 23 (29.5%) experienced RP‐ILD. Twenty‐two (22/34,64.7%) patients with RP‐ILD were positive for anti‐MDA5‐positive Ab and 1 case in antisynthetase Ab‐positive group. Among them, 20 patients (20/78, 25.6%) died, including 18 cases of anti‐MDA5 Ab‐positive dermatomyositis. Nineteen patients died from respiratory failure and 1 died from severe thrombocytopenia. The average survival of these patients was 3.1 months (1, 12), as shown in Table 1.

**Table 1:** Characteristics of study participants at diagnosis.

	Value	Range
Age (years, $\bar{x}$ ± s)	53.59 ± 13.10	(17–86)
Sex		
Male /Female	40/38 (1.05:1)	
Visit time (month, $\bar{x}$ ± s)	4.39 ± 5.88	(1–36)
Anti‐MDA5 Ab (+) (*n*)	34	
AAS (*n*)		
	Anti JO‐1Ab	13
	Ani‐EJ Ab	4
	Anti‐PL‐7 Ab	5
	Anti‐PL‐12 Ab	1
	Anti‐OJ Ab	2
	Anti‐SAE Ab	3
	Anti‐Mi‐2Ab	7
	Anti‐SRP Ab	3
	Anti‐TIFr‐1 Ab	6
	Anti‐Ro‐52Ab	47
	RP‐ILD	23
	ILD	62
Death	Anti‐MDA5 Ab‐positive group	18 (18/34)
	Anti‐MDA5 Ab‐negative group	2 (2/44)
Average survival of nonsurvivors	3.3 months	(1,12)

Abbreviations: Ab, antibody; MDA5, melanoma differentiation‐associated gene 5; RP‐ILD, rapidly progressive interstitial lung disease.

### 3.2 Demographic and clinical characteristics of dermatomyositis patients with or without anti‐MDA5 Ab

Clinical and laboratory features of 78 dermatomyositis patients with or without anti‐MDA5 Ab were examined. There was no significant difference in age and sex ratio between two groups (all *p* > .05). The incidence of arthritis (55.9% vs. 27.3%, *χ* ^2^ = 6.555, *p* = .019), fever (35.3% vs. 6.8%, *χ* ^2^ = 10.013, *p* = .003), skin ulcer (32.4% vs. 4.5%, *χ* ^2^ = 10.678, *p* = .002), Gottron's signs (91.2% vs. 38.6%, *χ* ^2^ = 22.369, *p* = .001), and RP‐ILD (64.7% vs. 2.3%, *χ* ^2^ = 39.65, *p* = .001) was significantly higher in patients with anti‐MDA5 Ab compared with patients without anti‐MDA5 Ab. In addition, the positivity of anti‐RO‐52 Ab (70.6% vs. 47.7%, *χ* ^2^ = 6.617, *p* = .012) was higher in patients with anti‐MDA5 Ab than that in without anti‐MDA5 Ab. The SF levels (1500.0 [658.80, 1844.0] vs. 352.20 [222.46, 437.0], *Z* = 5.188, *p* = .001) and γ‐GT (125.5 [61.0, 232.0] vs. 28 [16.0, 41.0], *Z* = 5.528, *p* < .001) were significantly higher, whereas serum CK (73.0 [42.0, 201.0] vs. 1333.0 [79.0, 8000.0], *Z* = −2.739, *p* = .006), albumin (32.51 ± 5.23 vs. 35.81 ± 5.88, *t* = −2.542, *p* = .013), and lymphocyte count (0.80 ± 0.36 vs. 1.45 ± 0.77, *t* = −4.717, *p* < .001) were lower in in the anti‐MDA5 Ab‐positive group. There was no significant difference in prevalence of heliotrope sign, myasthenia gravis, shawl sign, or dysphagia between two groups, as illustrated in Table 2.

**Table 2:** Demographic and clinical characteristics of dermatomyositis patients with or without anti‐MDA5 Ab.

	Anti‐MDA5 Ab‐positive group (*n* = 34)		Anti‐MDA5 Ab‐negative group (*n* = 44)	
	$\bar{x}$ ±s/M (IQR)	$\bar{x}$±s/M (IQR)	*t* /*χ* ^2^/*Z*	*p*
Age (year)	52.12 ± 12.1	55.27 ± 14.09	1.042	.301
Sex				
Male (%)	19 (55.9)	21 (47.7)		
Female	15 (34.1)	23 (67.6)	0.511	.502
Clinical characteristics (%)				
Arthritis	19 (55.9)	12 (27.3)	6.555	.019*
Fever	12 (35.3)	3 (6.8)	10.013	.003*
Gottron's signs	31 (91.2)	17 (38.6)	22.369	.001*
Skin ulcers	11 (32.4)	2 (4.5)	10.678	.002*
Mechanic's hand	2 (5.9)	6 (13.6)	1.253	.454
V sign	14 (41.2)	19 (43.2)	0.032	1.000
Heliotrope sign	21 (61.8)	17 (38.6)	4.106	.067
Shawl sign	10 (29.4)	16 (36.4)	0.417	.630
Myasthenia gravis	20 (58.8)	34 (77.3)	3.065	.067
Dysphagia	3 (8.8)	8 (18.2)	1.387	.331
Complications				
RP‐ILD	22 (64.7)	1 (2.3)	39.65	.001*
Laboratory				
Ferritin	1500.0 (658.80, 1844.0)	352.20 (222.46, 437.0)	5.188	.001*
KL‐6	777.5 (521.5,1812.0)	395.9 (212.0, 1763.0)	0.745	.456
CK	73.0 (42.0, 201,0)	1333.0 (79.0, 8000.0)	−2.739	.006*
γ‐GT	125.5 (61.0, 232.0)	28 (16.0, 41.0)	5.528	<.001*
Lymphocyte (×10^9^/L)	0.80 ± 0.36	1.45 ± 0.77	−4.717	<.001*
ALB	32.51 ± 5.23	35.81 ± 5.88	−2.542	.013*
Anti‐Ro‐52	26 (70.6)	21 (47.7)	6.617	.012*
Ab (+) (%)				

Abbreviations: γ‐GT, γ‐glutamyl transpeptidase; Ab, antibody; ALB, albumin; IQR, interquartile range; KL‐6, Krebs von den Lungen‐6; MDA5, melanoma differentiation‐associated gene 5; RP‐ILD, rapidly progressive interstitial lung disease; SF, serum ferritin.

* Statistical significance between different groups.

### 3.3 Clinical feature of anti‐MDA5 Ab‐positive DM patients with or without RP‐ILD

Among 34 anti‐MDA5 Ab‐positive dermatomyositis patients, 22 patients (64.75%) developed RP‐ILD. The complications of dermatomyositis patients with or without RP‐ILD were summarized in Table 3. The mean age and sex ratio were matched between two groups. The SF levels (1500.0 [658.80, 1844.0] vs. 352.20 [222.46, 437.0], *Z* = 5.188, *p* = .001), γ‐GT (134.0 [81.5, 204.5] vs. 123.0 [76.0, 189.0], *Z* = 3.136, *p* = .002), and the incidence of anti‐RO‐52 Ab (90.9% vs. 50.0%, *χ* ^2^ = 7.222, *p* = .013) were significantly higher in the anti‐MDA5 Ab‐positive group than those in the negative group. Lymphocyte count (0.58 ± 0.24 vs. 0.81 ± 0.33, *t* = 2.292, *p* = .029) was lower in anti‐MDA5 Ab‐positive group than that in anti‐MDA5 Ab‐negative group. There was no significant difference in the incidence of skin ulcer, Gottron's sign, fever and arthritis and the levels of KL‐6 and SP‐D between two groups (Table 3).

**Table 3:** Clinical feature of anti‐MDA5 Ab‐positive DM patients with or without RP‐ILD.

	Anti‐MDA5 Ab‐positive patients (*n* = 34)			
	RP‐ILD (*n* = 22)	noRP‐ILD (*n* = 12)	*t*/*Z*/*χ* ^2^	*p*
	$\bar{x}$ ± s/M (IQR)	$\bar{x}$ ± s/M (IQR)		
Age	54.73 ± 10.98	47.33 ± 13.06	−1.756	.089
Sex				
Male (%)	10 (45.5)	9 (75)		
Female	12 (54.5)	3 (25)	2.749	.152
Clinical characteristics (%)				
Gottron's	21 (95.5)	10 (83.3)	1.418	.279
Skin ulcers	10 (45.5)	1 (3.8)	4.889	.053
Fever	9 (40.9)	3 (25.0)	0.861	.294
Arthritis	13 (59.1)	6 (50.0)	0.26	.724
Laboratory				
Ferritin	1531.0 (1163.8, 2016.5)	584.9 (564.8, 1042.5)	2.664	.008^a^
KL‐6	854.0 (650.0, 2004.5)	331.0 (304.5, 584.0)	1.543	.123
CK	67.0 (52.0, 141.5)	79.0 (47.0, 438.5)	0.342	.732
γ‐GT	134.0 (81.5, 204.5)	123.0 (76.0, 189.0)	3.136	.002^a^
Lymphocyte (×10^9^/L)	0.58 ± 0.24	0.81 ± 0.33	2.292	.029^a^
Serum albumin	33.97 ± 3.99	31.74 ± 5.71	1.115	.257
Anti‐RO‐52	20 (90.9)	6 (50.0)	7.222	.013^a^
Ab (+) (%)				

Abbreviations: γ‐GT, γ‐glutamyl transpeptidase; Ab, antibody; ALB, albumin; IQR, interquartile range; KL‐6, Krebs von den Lungen‐6; MDA5, melanoma differentiation‐associated gene 5; RP‐ILD, rapidly progressive interstitial lung disease; SF, serum ferritin.

^a^ Statistical significance between different groups.

### 3.4 Clinical features of anti‐MDA5 Ab‐positive DM nonsurvivors and survivors with RP‐ILD

Of 34 anti‐MDA5‐positive DM patients, 16 patients survived (47.1%) and 18 (52.9%) died. Clinical features and laboratory data of surviving and dead anti‐MDA5 Ab‐positive DM patients were illustrated in Table 3. The mean age, disease duration, and sex ratio were matched between two groups. The SF levels (1544 (1447.32, 2089.0 vs. 584.9 [515.7,1500.0], *Z* = 2.096, *p* = .030) and the incidence of Anti‐Ro‐52 Ab ([16/18, 88.9%] vs. [9/16, 56.2%], *χ* ^2^ = 4.636, *p* = .031) were significantly higher in death group than those in the survival group. Lymphocyte count (0.56 ± 0.25 vs. 0.78 ± 0.30, *t* = 2.252, *p* = .031) was lower in nonsurvivors. The incidence of fever, arthritis, skin ulcer, and the levels of γ‐GT, KL‐6, and SP‐D did not differ between the two groups.

In the anti‐MDA5‐positive group, 22 cases were treated with corticosteroid combined with cyclophosphamide, cyclosporine, or tacrolimus (12 cases died and 10 cases survived), and the remaining cases were treated with corticosteroid combined with tofacitinib (10 mg/day) in 5 cases (1 case died and 4 cases survived), corticosteroids combined with abatacept in 2 cases (1 case died and 1 case survived), and corticosteroids combined with CTX in 5 case (4 patients died and 1 survived). The average dose of corticosteroid was 60 mg/day and only one patient was treated with 500 mg methylprednisolone, as illustrated in Table 4.

**Table 4:** Clinical features of anti‐MDA5 Ab‐positive DM nonsurvivors and survivors with RP‐ILD.

	Anti‐MDA5 Ab‐positive patients (*n* = 34)			
	Non‐survivors (*n* = 18)	Survivors (*n* = 16)	*t*/*χ* ^2^/*Z*	*p*
	*x* ± s/M (IQR)	$\bar{x}$ ± s/M (IQR)		
Age	55.39 ± 10.86	48.44 ± 12.69	−1.712	.095
Duration (m)	2.78 ± 2.51	2.28 ± 2.03	−0.630	.533
Sex (%)				
Male	9 (50%)	10 (62.5%)		
Female	9 (50%)	6 (37.5%)	0.537	.510
Clinical characteristics (%)				
Skin ulcers	8 (44.4%)	3 (17.6%)	2.913	.146
Gottron sign	17 (94.4%)	14 (82.4%)	1.263	.338
Fever	8 (44.4%)	4 (23.5%)	1.697	.289
Arthritis	11 (61.1%)	9 (52.9%)	0.238	.738
Laboratory				
L (10^−9^/L)	0.56 ± 0.25	0.78 ± 0.30	2.252	.031^a^
Ferritin	1544 (1447.32, 2089.0)	584.9 (515.7, 1500.0)	2.096	.030^a^
KL‐6	881.0 (718.0, 1030.0)	480.0 (304.5, 741.5)	−1.563	.115
CK	67 (52, 468.5)	79 (37, 156)	.276	.782
γ‐GT	134.0 (31.5, 210.5)	123 (89, 232)	1.639	.101
AKP	117 (80, 149)	86 (79, 146)	0.207	.836
ALB	33.24 ± 5.01	32.59 ± 5.51	0.192	.849
Positive rate of Anti‐Ro‐52 (%)	16 (88.9%)	9 (56.2%)	4.636	.031^a^
Treatment				
P + CTX + TAk/CsA	12 (66.7%)	10 (62.5%)		
P + Tofacitinib	1 (1/18)	4 (4/16)		
P + Abatacept	1 (1/18)	1 (1/16)		
P + CTX	4 (4/18)	1 (1/16)		

Abbreviations: γ‐GT, γ‐glutamyl transpeptidase; Ab, antibody; ALB, albumin; IQR, interquartile range; KL‐6, Krebs von den Lungen‐6; MDA5, melanoma differentiation‐associated gene 5; RP‐ILD, rapidly progressive interstitial lung disease.

^a^ Statistical significance between different groups.

### 3.5 Risk of death in anti‐MDA5 Ab‐positive group and its diagnostic value for death

**Figure 1:**
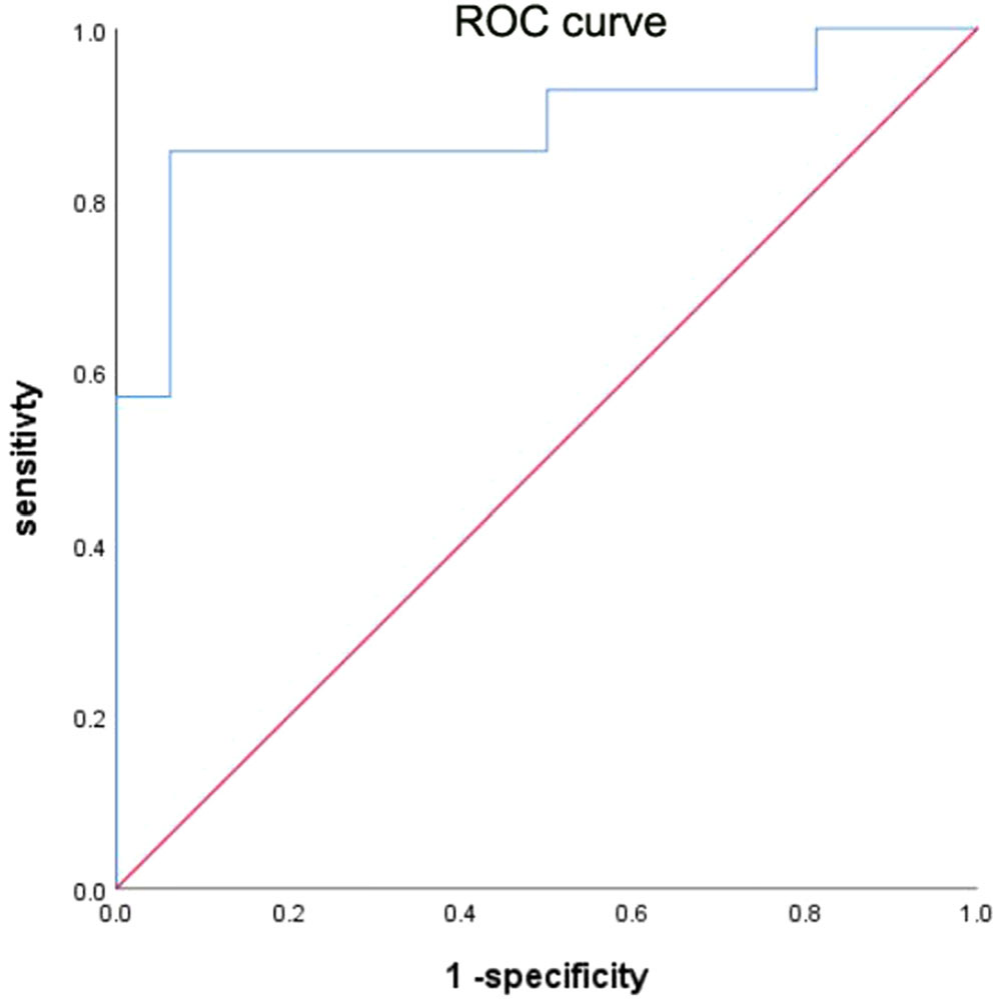
The sensitivity and specificity of the receiver operating characteristic curve analysis.

In the anti‐MDA5‐positive group, death or not (1 = survival and 2 = death) was taken as the dependent variable and the variables with *p* < .05 in Table 4 were considered as the independent variables (noncontinuous variable 1 = negative and 2 = positive) for logistic regression analysis. Lymphocyte count was regarded as the risk factor for death. As illustrated in Figure 1, ROC curve showed that the area under the curve was 0.888, *p* < .001, 95% CI (0.756, 1.000), the sensitivity was 85.7, the specificity was 93.8, and the Youden's index was 0.795, as shown in Table 5.

**Table 5:** Risk factors for death in anti‐MDA5 Ab (+) group.

	B	SE	Wald	*p*	OR	95% CI
Constant	4.009	1.707	5.514	0.019	55.080	‐
Lymphocyte	−6.904	2.666	6.708	0.01	0.001	0.000‐0.186
Serum ferrin	0.000	0.000	0.191	0.662	1.000	0.999‐1.001
An‐RO‐52 Ab	0.384	1.342	0.082	0.775	1.469	0.106‐20.382

Abbreviations: 95% CI, 95% confidence interval; MDA5, melanoma differentiation‐associated gene 5; OR, odds ratio.

## 4 DISCUSSION

Dermatomyositis is an inflammatory myopathy with strong heterogeneity. Patients with anti‐MDA5 Ab‐positive dermatomyositis have poor prognosis and high mortality within 6 months.^2^, ^5^ The median survival of nonsurvivors was 3.3 months in this study. Studies have reported that clinical characteristics in dermatomyositis patients with anti‐MDA5 Ab compared with anti‐MDA5‐negative patients. Skin ulcer and arthritis^14^, ^15^ have been reported to be associated with the presence of anti‐MDA5 Ab. The incidence of RP‐ILD^16^ is significantly higher in the anti‐MDA5 Ab‐positive group than in the Ab‐negative group. Anti‐MDA5 Ab‐positive patients with ILD or RP‐ILD are characterized by higher SF and lower CK. Consistent with previous reports, our study confirms that the incidence of arthritis, skin ulcer, Gottron's signs, and RP‐ILD was markedly higher in dermatomyositis patients with anti‐MDA5 Ab, whereas mechanic hand, heliotrope rash, V sign, and shawl sign did not differ between the two groups. The lymphocyte count in peripheral blood, serum albumin, and the level of CK was lower than those in the negative group. Further studies showed that the levels of γ‐GT, SF, and the positive of anti‐RO‐52kD in patients with RP‐ILD in anti‐MDA5 Ab‐positive group were higher than those without RP‐ILD, whereas the lymphocyte count in patients with RP‐ILD was lower than those without RP‐ILD. High SF and low CK levels are important predictors of RP‐ILD in dermatomyositis.^2^, ^3^, ^16^ In this study, the SF level was not only significantly higher in the anti‐MDA5 Ab‐positive group but also significantly higher in the anti‐MDA5 Ab‐positive group with RP‐ILD than in those without RP‐ILD. Considerations may be related to differences in the study population. The subjects of this study were dermatomyositis patients with RP‐ILD in the anti‐MDA5 Ab‐positive group, whereas the Hamaguchi^2^ study involved all dermatomyositis patients. In addition, previous study has found some potential risk factors related to RP‐ILD and prognosis, such as high fever as a risk factor for RP‐ILD.^17^ In this study, there were more fever patients in the anti‐MDA5 Ab‐positive group than in the negative group. In another study of 1269 patients with idiopathic interstitial pneumonia, Zisman et al.^18^ found that hypoalbuminemia was independently associated with higher mortality, the possible reason was that albumin acts as a protective factor that could inhibit endothelial cell apoptosis, prevents the production of oxygen free radical, and reduces platelet aggregation. As large amounts of cytokines and inflammatory medium were produced in the course of the ILD disease, which could lead to a decrease in hepatic albumin synthesis, pulmonary fibrosis could be activated and progressed because of the protective effect of albumin was diminished. This study only found that the albumin level was lower in anti‐MDA5 Ab‐positve group than that in negative group, but there was no statistical difference in the occurrence of RP‐ILD and the survival and death groups, which may be related to the small number of cases after grouping. Gono et al.^7^ analyzed clinical characteristics of 14 dermatomyositis patients with anti‐MDA5 Ab‐positive and anti‐synthetase Ab‐positive, and found that the former had lower CK and higher SF level. There was no difference in KL‐6 between two groups, which was consistent with our study. However, Gono et al.^7^ found that γ‐GT level was higher in dead patients than that in survivors. In this study, γ‐GT was higher in the death group than in the survival group, but there was no statistical difference. γ‐GT was a liver specific enzymatic marker. Liver may be one of the organs involved in anti‐MDA5 Ab‐positive dermatomyositis, as well as skin and lung was as one of the organs involved in anti‐MDA5 Ab‐positive dermatomyositis.^7^

Abs to SSA antigen (Ro52) have been described histologically as markers of Sjögren syndrome. However, clinical studies have shown that anti‐SSA/Ro52 Abs were associated with ILD^19^, ^20^ and DM patients with anti‐MDA5 and anti‐SSA‐52 double positive had a high mortality rate and poor prognosis.^19^ The prevalence of anti‐SSA/Ro52 Ab in rheumatic diseases suggested that this Ab was related to the phenotype or clinical manifestations of connective tissue diseases. Epitope mapping studies^21^ shown that SSA/Ro52 Abs bound to one peptide in the Ro52 helical region were associated with the incidence and severity of ILD in rheumatic diseases. The results of this study showed that the positive rate of anti‐SSA‐52 was high in the group of dead patients, but no correlation was found in further regression analysis. In this study, anti‐SSA/RO52 Ab‐positive in dermatomyositis patients with anti‐MDA5 Abs reveals a dermatomyositis subgroup with poor prognosis. These findings indicate that the role of anti‐Ro52 in infection response is probably correlated with anti‐MDA5 pathogenesis.^19^

In this study, lymphocyte count was lower in the anti‐MDA5 Ab‐positive group and further study found that the lymphocyte count in the patients with MDA5 and RP‐ILD was also lower than that in the patients without RP‐ILD. Logistic regression analysis showed that the decrease of lymphocyte count was one of the risk factors of death. More research is needed on how lymphocytes, particularly T lymphocytes, work in patients with MDA5‐positive dermatomyositis. An early study reported that patients with DM could develop lymphopenia, characterized by low peripheral CD4^+^ and CD8^+^ T‐cell and B‐cell absolute counts before initial treatment and a significant increase in lymphocyte counts after treatment.^22^ Shu et al.^23^ found that the autophagy of T lymphocytes in dermatomyositis patients was inhibited, leading to the upregulation of lymphocyte apoptosis and lymphopenia. Studies^24^, ^25^, ^26^ speculated that lymphocytes migrated to the lungs to participate in local immune responses, resulting in a decrease in peripheral blood lymphopenia. Yu et al.^25^ found that the decrease of lymphocyte count was closely related to RP‐ILD and considered that the onset of MDA5‐positive dermatomyositis might be correlated with the medical history before infection. Therefore, it is speculated that infection with viruses or other pathogens can also lead to lymphocyte depletion in patients with MDA5positive dermatomyositis. In the MDA5‐positive dermatomyositis group, in addition to T‐cell abnormalities, the increased level of B2 (CD5‐CD19^+^) cells is also a risk factor for RP‐ILD, which will be further studied. Elevated lymphocytes are considered to be sensitive to glucocorticoid and immunosuppressive therapy, whereas low lymphocyte counts are not sensitive to treatment. It is speculated that this may be one of the reasons accounting for ineffective treatment to various therapies and high mortality rate of anti‐MDA‐5 Ab‐positive patients.

Patients with RP‐ILD who are anti‐MDA5 Ab‐positive have poor prognosis, high mortality, and poor response to treatment; therefore, it is important to identify simple and easy predictors. This study suggested that anti‐MDA5 Ab‐positive dermatomyositis patients had a higher incidence of RP‐ILD and a higher mortality than anti‐MDA5 Ab‐negative dermatomyositis patients. Anti‐MDA5 Ab combined with RP‐ILD obtained high SF, low lymphocyte count and high positive rate of anti‐RO‐52 Ab. Multivariate regression analysis showed that the decrease of lymphocyte count was a risk factor for death in dermatomyositis patients with positive anti‐MDA5 Ab.

### 4.1 Study limitations

Several limitations have to be acknowledged in this study. First, the titer of anti‐MDA5 Ab was not analyzed. Second, the lymphocyte subsets in RP‐ILD patients positive for anti‐MDA5 Ab were not investigated. Third, the sample size was relatively small. Fourth, patients with different stages of dermatomyositis were not included and analyzed.

## 5 CONCLUSION

Taken together, patients with anti‐MDA5 Ab‐positive dermatomyositis are prone to suffering from RP‐ILD and the decrease in lymphocyte count is an important risk factor for complicated with RP‐ILD and patient death, which may be a convenient and feasible predictor for anti‐MDA5 Ab‐positive dermatomyositis in Chinese population.

## AUTHOR CONTRIBUTIONS

**Li Lian**: Conceptualization; data curation; investigation; methodology; writing—original draft; writing—review & editing. **Jing‐jing Tong**: Conceptualization; data curation; investigation; methodology; project administration. **Sheng‐qian Xu**: Investigation; methodology; writing—original draft; writing—review & editing.

## CONFLICT OF INTEREST STATEMENT

The authors declare no conflict of interest.

## Data Availability

The data sets used and/or analyzed during the current study available from the corresponding author on reasonable request.
